# Retraction Note: Peri-implantitis-associated methanogens: a preliminary report

**DOI:** 10.1038/s41598-024-51888-w

**Published:** 2024-01-16

**Authors:** Souad Belkacemi, Anthony Mazel, Delphine Tardivo, Patrick Tavitian, Grégory Stephan, Giancarlo Bianca, Elodie Terrer, Michel Drancourt, Gérard Aboudharam

**Affiliations:** 1 Aix-Marseille Univ, IRD, MEPHI, IHU Méditerranée-Infection, Marseille, France; 2 UFR Odontologie, Aix-Marseille Université, Marseille, France; 3 Private practice Marseille France, Marseille, France

Retraction of: *Scientific Reports* 10.1038/s41598-018-27862-8, published online 21 June 2018

Editors have retracted this Article.

After publication of this paper concerns about ethical oversight of this study were brought to the attention of the Editors. The Authors were not able to provide documentation of approval from an appropriate ethics committee for the work reported.

Michel Drancourt did not explicitly state whether or not he agreed with this retraction. The remaining Authors did not respond to correspondence from the Editors about this retraction.

